# Role of “dual-personality” fragments in HEV adaptation—analysis of Y-domain region

**DOI:** 10.1186/s43141-021-00238-8

**Published:** 2021-10-12

**Authors:** Zoya Shafat, Anwar Ahmed, Mohammad K. Parvez, Shama Parveen

**Affiliations:** 1 Centre for Interdisciplinary Research in Basic Sciences, Jamia Millia Islamia, New Delhi, India; 2 Centre of Excellence in Biotechnology Research, College of Science, King Saud University, Riyadh, Saudi Arabia; 3 Department of Pharmacognosy, College of Pharmacy, King Saud University, Riyadh, Saudi Arabia

**Keywords:** Y-domain region (YDR), Protein structure, Protein disorder, Protein-binding propensity, Nucleotide-binding propensity, Phosphorylation, Molecular function

## Abstract

**Background:**

Hepatitis E is a liver disease caused by the pathogen hepatitis E virus (HEV). The largest polyprotein open reading frame 1 (ORF1) contains a nonstructural Y-domain region (YDR) whose activity in HEV adaptation remains uncharted. The specific role of disordered regions in several nonstructural proteins has been demonstrated to participate in the multiplication and multiple regulatory functions of the viruses. Thus, intrinsic disorder of YDR including its structural and functional annotation was comprehensively studied by exploiting computational methodologies to delineate its role in viral adaptation.

**Results:**

Based on our findings, it was evident that YDR contains significantly higher levels of ordered regions with less prevalence of disordered residues. Sequence-based analysis of YDR revealed it as a “dual personality” (DP) protein due to the presence of both structured and unstructured (intrinsically disordered) regions. The evolution of YDR was shaped by pressures that lead towards predominance of both disordered and regularly folded amino acids (Ala, Arg, Gly, Ile, Leu, Phe, Pro, Ser, Tyr, Val). Additionally, the predominance of characteristic DP residues (Thr, Arg, Gly, and Pro) further showed the order as well as disorder characteristic possessed by YDR. The intrinsic disorder propensity analysis of YDR revealed it as a moderately disordered protein. All the YDR sequences consisted of molecular recognition features (MoRFs), i.e., intrinsic disorder-based protein–protein interaction (PPI) sites, in addition to several nucleotide-binding sites. Thus, the presence of molecular recognition (PPI, RNA binding, and DNA binding) signifies the YDR’s interaction with specific partners, host membranes leading to further viral infection. The presence of various disordered-based phosphorylation sites further signifies the role of YDR in various biological processes. Furthermore, functional annotation of YDR revealed it as a multifunctional-associated protein, due to its susceptibility in binding to a wide range of ligands and involvement in various catalytic activities.

**Conclusions:**

As DP are targets for regulation, thus, YDR contributes to cellular signaling processes through PPIs. As YDR is incompletely understood, therefore, our data on disorder-based function could help in better understanding its associated functions. Collectively, our novel data from this comprehensive investigation is the first attempt to delineate YDR role in the regulation and pathogenesis of HEV.

**Supplementary Information:**

The online version contains supplementary material available at 10.1186/s43141-021-00238-8.

## Background

Hepatitis E virus (HEV) is a non-enveloped RNA virus of the family *Hepeviridae* [1]. HEV is the major causative agent of acute hepatitis worldwide. Largely, the infection is asymptomatic in the general population; however, HEV can lead to severe infections in pregnant women, such as fulminant hepatic failure with a high mortality rate (20–30%) [2]. Recently, it has been estimated that around 939 million individuals across the globe have experienced past HEV infection and around 15–110 million of the population are still undergoing or experiencing recent infections [3].

HEV is currently segregated into eight genotypes (GT 1 to GT 8). GT 1 and GT 2 infect humans and are mainly transmitted through contaminated water causing acute hepatitis while GT 3 and GT 4 strains have an expanded range of hosts which includes humans, rabbits, wild boars, and pigs [4–7] and are the cause of chronic HEV infections, especially in organ transplant patients [8, 9]. However, studies have reported the isolation of other strains of HEV from specific hosts, such as GT 5 and GT 6 from wild boars in Japan [10, 11], GT 7 from dromedary camels [12], and HEV-8 from Bactrian camels [13]. Consumption of uncooked/undercooked or raw animal meat products is regarded as the main cause of sporadic cases of HEV in developed countries [14]. Due to the continuous increase in the number of newly discovered strains and expanding host range, the implications of HEV on the health of humans remain doubtful [14]. This further complicates the transmission and the risk of HEV infection. Besides water- and food-mediated transmission routes, blood-borne transmission has also been reported in patients receiving organ transplantation [15]. Additionally, person-to-person transmission has recently been reported [16]. Additionally, evidence has indicated that pet animals including cat, dog, rabbit, and horse act as accidental hosts in the transmission of HEV to humans [17, 18]. Thus, HEV has become a global health burden in both the developing as well as developed countries and therefore requires urgent attention to design its preventive measures. Anti-HEV IgG antibody is considered as the marker for persons who have experienced past infection as it usually persists for many years [19, 20]. In contrary to this, anti-HEV IgM antibody is regarded as a marker for the ongoing or recent infection in individuals as it is short-lived (up to few months). HEV RNA detection is considered as the bona fide marker for the active ongoing infection in the population.

The HEV genome is systematized into three partially over-lapped ORFs (ORF1, ORF2, and ORF3) [21]. The largest ORF1 encodes the nonstructural proteins required for the viral replication [22, 23]. ORF2 encodes the viral capsid protein [24, 25], and ORF3 encodes a protein, which has regulatory functions [26–28]. The ORF1 nonstructural YDR (Y-domain region) is the second domain at 5′ end and is situated between the methyltransferase (MTase) and putative cysteine protease (PCP) domains [29, 30]. The HEV YDR critical residue indispensability was first reported by Parvez [30]. This study has suggested the presence of universally conserved residues (L_410_, S_412_, and W_413_) in the predicted YDR alpha-helix homolog (LYSWLFE). These critical residues have been demonstrated to play crucial role in the RNA replication of the virion [30]. It was also determined that mutations in the highly conserved cysteine dyad (C_336_–C_337_), attributed to membrane binding, completely abolished RNA replication. Such functional and/or structural integrity clearly suggests YDR essentiality in replication of HEV that might embody common principles of YDR and cytoplasmic membrane interaction [30]. Although a recent study has proposed the role of YDR in HEV replication by suggesting the essentiality of two conserved motifs (putative palmitoylation site and an alpha-helical segment) in the HEV life cycle [30], a direct correlation between the function of YDR conserved segments and viral adaptation has not been discovered. Thus, we attempted to delineate the role of YDR in viral adaptation.

The present study analyzed the structurally “unknown” regions (i.e., a fraction of a proteome that has no detectable similarity to any PDB structure) of the HEV YDR. This fraction we call it as the “dark proteome.” These disordered protein regions exist as extremely active ensembles that are rapidly interconvertible under different physiological conditions [31–33]. Due to the occurrence of a peculiar phenomenon, i.e., binding of several disordered regions to one ligand or vice versa (one disordered region binds to many partners), the intrinsic disordered regions are utilized in protein–protein interactions [34, 35]. Thus, the intrinsic disordered regions in proteins are considered as potential drug targets due to disordered to ordered transition state upon drug binding [36]. The current study reports analysis on the disordered side of HEV YDR using a combination of different computational methods to check the occurrence of disordered regions in order to gain insights into their disorder-related functions. As unstructured regions in viruses are strongly associated with virulence, thus, the identification of protein functions related to disorder will shed some light on the role of YDR in HEV adaptation.

## Methods

### Sequences

The protein sequences of HEV YDR were obtained from the GenBank. The individual protein sequence considered for the present analysis included a total of eight study sequences. The individual sequence included different genotypes, i.e., GT 1–GT 8 as currently eight genotypes have been recognized in HEV. The obtained sequences were accumulated in such a way that they encompassed different host organisms (human, swine, wild boar, and camel). Thus, we carried out multiple predictions of these eight study sequences by computational methods and comparative analyses were performed.

### Structural analysis

The 3D models of HEV YDR sequences were predicted using Phyre2 (Protein Homology/AnalogY Recognition Engine) server (http://www.sbg.bio.ic.ac.uk/~phyre2/html/page.cgi?id=index) [37] and analyzed.

### Amino acid distribution

The amino acid composition of the individual sequences of HEV YDR was computed and thoroughly analyzed. The analysis was conducted using the online webserver Expasy ProtParam (https://web.expasy.org/protparam/).

### Protein disorder and flexibility prediction

Intrinsically disordered regions (IDRs) of the YDR sequences were predicted using the PONDR® (Predictor of Natural Disordered Regions) at its default settings. Multiple predictors such as members of the PONDR® family including PONDR®VLS2 [38], PONDR®VL3 [39], and PONDR® VLXT [40] were exploited to predict the intrinsic disorder predisposition in YDR. This bioinformatics tool predicts the residues or regions which fail in propensity for an ordered structure formation. The protein residues with predicted scores between 0.2 and 0.5 were considered as flexible, while the residues which had scores exceeding the 0.5 threshold value were predicted as intrinsically disordered ones.

### Protein-binding region prediction

MoRFpred [41] online bioinformatics predictor was used to identify the protein–protein interaction regions within the HEV YDR sequences. This webserver is designed to recognize the protein Molecular Recognition Features (MoRFs). The residues which scored above the threshold value of 0.5 were considered as MoRF regions.

### Nucleotide-binding region prediction

Various online servers are available to predict the RNA- and DNA-binding regions within the YDR sequences. DisoRDPbind webserver predicts the RNA-, DNA-, and protein-binding residues located in the intrinsically disordered region of proteins. DRNApred webserver provides a sequence-based prediction of DNA- and RNA-binding residues within proteins. PPRInt webserver predicts the RNA-interacting amino acid residues in the given sequence. Thus, these tools were used in combination to predict the RNA- and DNA-interacting residues within the HEV YDR sequences.

#### RNA-binding residue prediction

For RNA-binding residue identification, we used a combination of three webservers, i.e., DisoRDPbind [42], DRNApred [43], and PPRInt [44].

#### DNA-binding residue prediction

For DNA-binding residue identification, we used a combination of two webservers, i.e., DisoRDPbind [42] and DRNApred [43] webservers.

### Phosphorylation prediction

The phosphorylated Ser, Thr, and Tyr residues in HEV YDR sequences were predicted using the online tool DEPP (Disorder enhanced phosphorylation prediction) (http://www.pondr.com/cgi-bin/depp.cgi). The disorder information is used by the DEPP algorithm to improve the discrimination between phosphorylation and non-phosphorylation sites. The accuracy of DEPP reaches 76.0 ± 0.3%, 81.3 ± 0.3%, and 83.3 ± 0.3% for Ser, Thr, and Tyr respectively.

### Structure-based function prediction

As HEV exhibits a broad-host range, thus, HEV YDR 3D structural models were generated using YDR sequences obtained from different host organisms. The probable molecular functions were predicted using the COFACTOR algorithm [45, 46]. The analysis was conducted using the sequences AF444002 (HEV), JF443720 (human), GU119961 (swine), AB222182 (wild boar), and KJ496143 (camel).

## Results

**Fig. 1:**
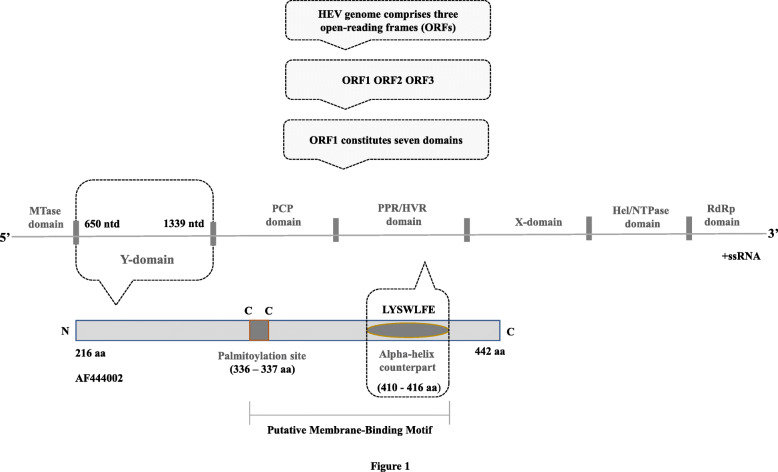
Diagrammatic representation of hepatitis E virus nonstructural polyprotein (ORF1) domain, showing the Y-domain. The ORF1 constitutes seven domains, i.e., MTase, methyltransferase; Y, undefined; PCP, papain-like cysteine protease; P/HVR, proline-rich/hypervariable region; X, Macro; Hel/NTPase, helicase/nucleotide triphosphatase; and RdRp, RNA-dependent RNA polymerase. The Y-domain region (YDR) is of 228 amino acids in length (650–1339 nucleotides) and consists of a potential palmitoylation site (C_336_C_337_) and an alpha-helix segment (L_410_Y_411_S_412_W_413_L_414_F_415_E_416_). These segments are found to be indispensable for cytoplasmic membrane binding and are highly conserved within HEV genotypes

The HEV genome comprises three ORFs (ORF1, ORF2, and ORF3): The ORF1 consists of seven domains, i.e., MTase, methyltransferase; Y, undefined; PCP, papain-like cysteine protease; P/HVR, proline-rich/hypervariable region; X, macro; Hel/NTPase, helicase/nucleotide triphosphatase; and RdRp, RNA-dependent RNA polymerase. The Y-domain region (YDR) is of 228 amino acids in length (650–1339 nucleotides) and consists of a potential palmitoylation site (C_336_C_337_) and an alpha-helix segment (L_410_Y_411_S_412_W_413_L_414_F_415_E_416_). These segments are found to be indispensable for cytoplasmic membrane binding and are highly conserved within HEV genotypes. The YDR of HEV GT 1 (accession number: AF444002) is represented in Fig. 1.

### Retrieval of sequences

The YDR sequences were analyzed to assess its disorder-based binding functions, using different computational approaches. The list of sequences considered for the present analysis is listed as supplemental material (S1 Table).

### Structural annotation

Comprehensive analyses of protein structures provide a detailed understanding of its function conformation in terms of amino acid sequence and composition. Thus, the YDR structure was examined thoroughly using a web portal for protein modeling and analysis. The predicted 3D models for YDR sequences were generated through the homology modeling approach (S1A–H Figure). Three states of secondary structure: helix (H; includes alpha-, pi-, and 3_10-helix), (beta-)strand (E = extended strand in beta-sheet conformation of at least two residues length), and loop (L) were identified in YDR models. The results in the YDR sequences showed the dominance of coils followed by helices and strands (S1A–H Figures). It was found that connectivity between secondary structure elements was made by long loops, called the coiled region. Additionally, in the obtained YDR models, the amino acid residues that were found to be missing indicated the presence of high conformational flexible regions (S1A–H Figure).

### Analysis of amino acid distributions

**Fig. 2:**
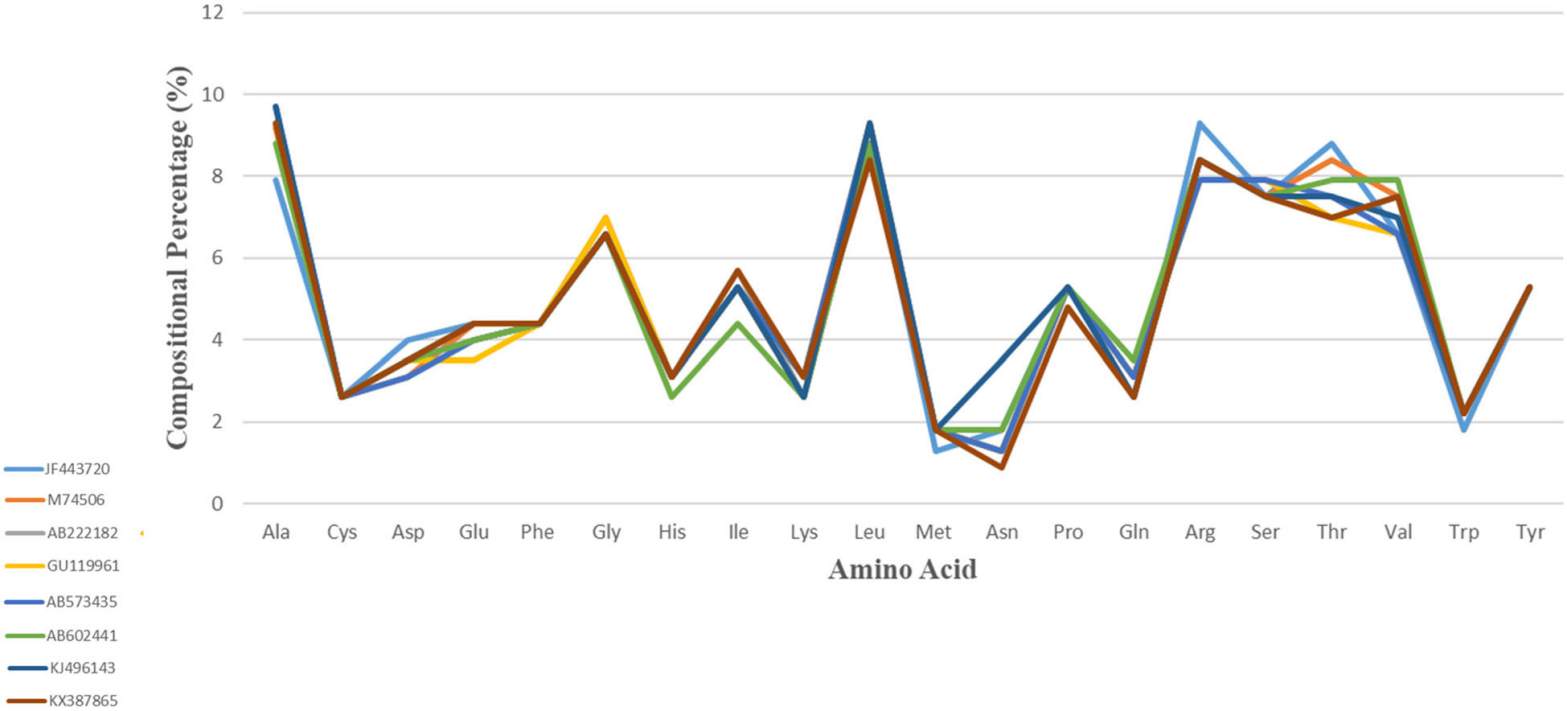
Depiction of amino acid percentage composition in the YDR sequences considered for the study: (A) JF443720 (GT 1), (B) M74506 (GT 2), (C) AB222182 (GT 3), (D) GU119961 (GT 4), (E) AB573435 (GT 5), (F) AB602441 (GT 6), KJ496143 (GT 7), and (H) KX387865 (GT 8)

The amino acid composition was thoroughly examined to identify the characteristic residue features in the YDR. The predicted amino acid percentages in YDR sequences are mentioned in Table 1 and Fig. 2.

**Table 1:** The predicted amino acid percentages of YDR in hepatitis E viruses

AA	JF443720	M74506	AB222182	GU119961	AB573435	AB602441	KJ496143	KX387865
Ala	7.9	9.17	9.3	9.3	9.7	8.8	9.7	9.3
Cys	2.6	2.6	2.6	2.6	2.6	2.6	2.6	2.6
Asp	4.0	3.1	3.5	3.5	3.1	3.5	3.5	3.5
Glu	4.4	4.4	4.0	3.5	4.0	4.0	4.4	4.4
Phe	4.4	4.4	4.4	4.4	4.4	4.4	4.4	4.4
Gly	6.6	7.0	6.6	7.0	6.6	6.6	6.6	6.6
His	3.1	3.1	3.1	3.1	3.1	2.6	3.1	3.1
Ile	5.3	5.3	5.7	5.3	5.3	4.4	5.3	5.7
Lys	2.6	2.6	2.6	3.1	3.1	2.6	2.6	3.1
Leu	8.8	8.8	8.8	9.3	9.3	8.8	9.3	8.4
Met	1.3	1.8	1.8	1.8	1.8	1.8	1.8	1.8
Asn	1.8	1.3	1.3	1.3	1.3	1.8	3.5	0.9
Pro	5.3	5.3	5.3	5.3	5.3	5.3	5.3	4.8
Gln	2.6	3.1	3.1	3.1	3.1	3.5	2.6	2.6
Arg	9.3	8.4	8.4	7.9	7.9	8.4	8.4	8.4
Ser	7.5	7.5	7.5	7.9	7.9	7.5	7.5	7.5
Thr	8.8	8.4	7.0	7.0	7.5	7.9	7.5	7.0
Val	6.6	7.5	7.5	6.6	6.6	7.9	7.0	7.5
Trp	1.8	2.2	2.2	2.2	2.2	2.2	2.2	2.2
Tyr	5.3	5.3	5.3	5.3	5.3	5.3	5.3	5.3

Note 1: The amino acid values are represented as percentages

Note 2: JF443720 (GT 1); M74506 (GT 2); AB222182 (GT 3); GU119961 (GT 4); AB573435 (GT 5); AB602441 (GT 6); KJ496143 (GT 7); KX387865 (GT 8)

### Categorization of protein structure

Unexpectedly, the presence of both hydrophobic and polar residues was favored in YDR sequences. The amino acids on the basis of their relative abundance ratios are clustered into three major classes: ordered (O), disordered (D), and dual personality (DP) [47].

- The first group constitutes the very small (Ala, Gly, Ser) as well as few hydrophilic (Glu, Lys) amino acids. These amino acids are prevalent in D fragments, while deficient in O fragments.
- The second group comprises mostly hydrophilic amino acids (Asp, Thr, Gln, Asn, Pro, and Arg). Most of these amino acids show a higher preference towards DP fragments.
- The third group constitutes the mostly hydrophobic amino acids (Ile, Phe, Tyr, His, Met, Cys, and Trp). These amino acids are deficient in D fragments while showing abundance in O fragments.

The considered study sequences of YDR for our analysis were observed with a higher preference towards both ordered (Leu, Phe, Tyr, Val) and disordered amino acid residues (Ala, Arg, Gly, Pro, Ser) [48–54] (Fig. 2). Our results thus indicated the abundance of both order-promoting and disorder-promoting amino acid residues in YDR sequences, which clearly revealed the characteristics of protein hybrids, i.e., proteins having both intrinsically disordered regions (IDPRs) and structured regions. Furthermore, the abundance of signature hydrophobic amino acid residues such as Thr, Arg, Gly, and Pro revealed that YDR possessed the characteristics of “Dual Personality” (DP) fragments, i.e., the prevalence of order as well as disorder characteristics [47]. These DP protein segments exist either in the ordered (O) or in the disordered (D) states and thus are designated as DP fragments. Therefore, DP is more rigid (ordered) in some conditions while more flexible (disordered) in others. Due to this fact, DP fragments are marginally stable in both the buried and exposed parts of the protein model [47].

### Analysis of protein disorder and flexibility

#### PONDR

**Fig. 3:**
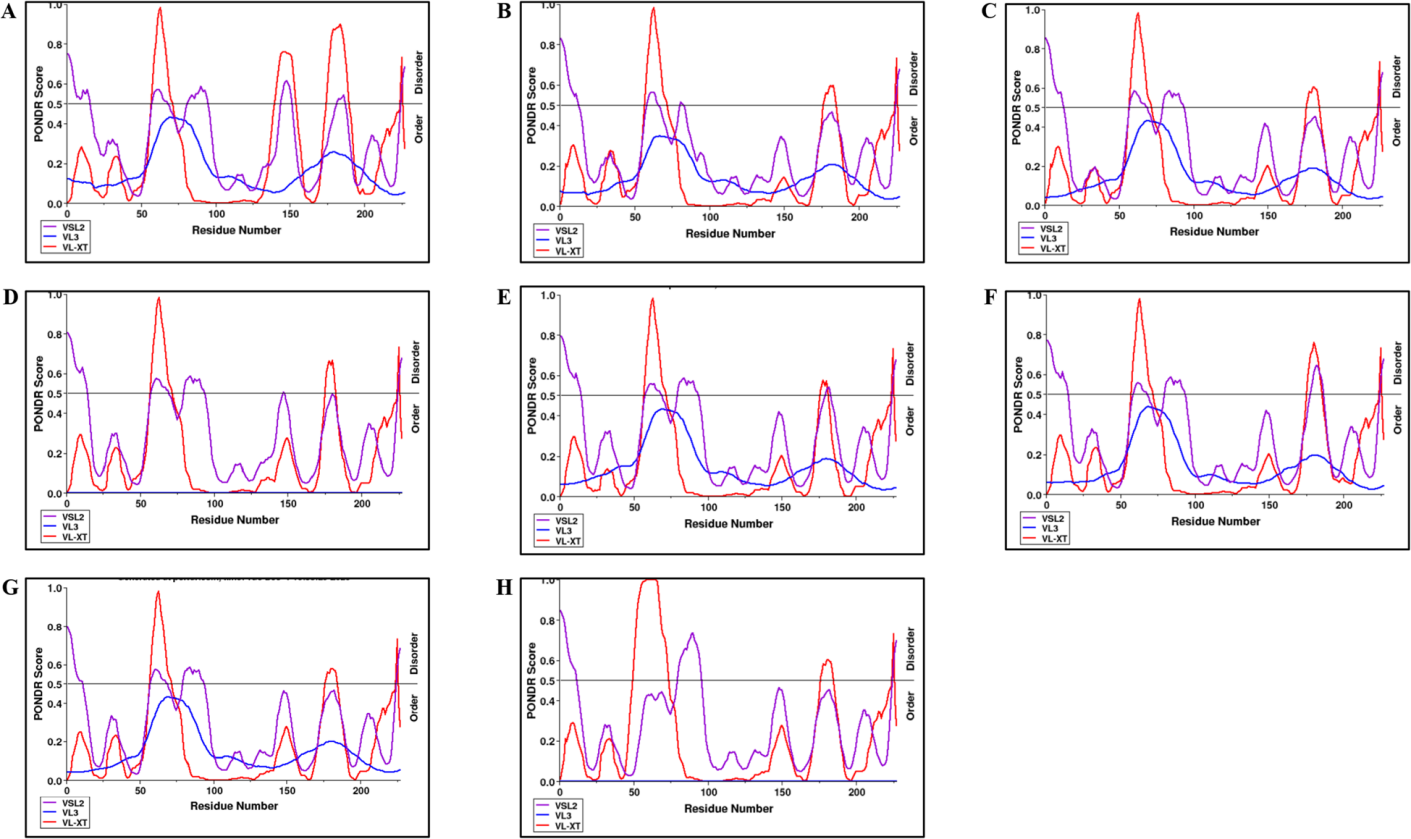
Analysis of intrinsic disorder predisposition of HEV YDR. (**A**) JF443720 (GT 1); (**B**) M74506 (GT 2); (**C**) AB222182 (GT 3); (**D**) GU119961 (GT 4); (**E**) AB573435 (GT 5); (**F**) AB602441 (GT 6); KJ496143 (GT 7); and (**H**) KX387865 (GT 8). Graphs **A**–**H** represent the intrinsic disorder profiles of YDR sequences of HEV. Disorder probability was calculated using three members of the family PONDR (Prediction of Natural Disordered Regions), i.e., VLXT, VL3, and VSL2. A threshold value of 0.5 was set to distinguish between ordered and disordered regions along the genome (dashed line). Regions above the threshold are predicted to be disordered

**Fig. 4:**
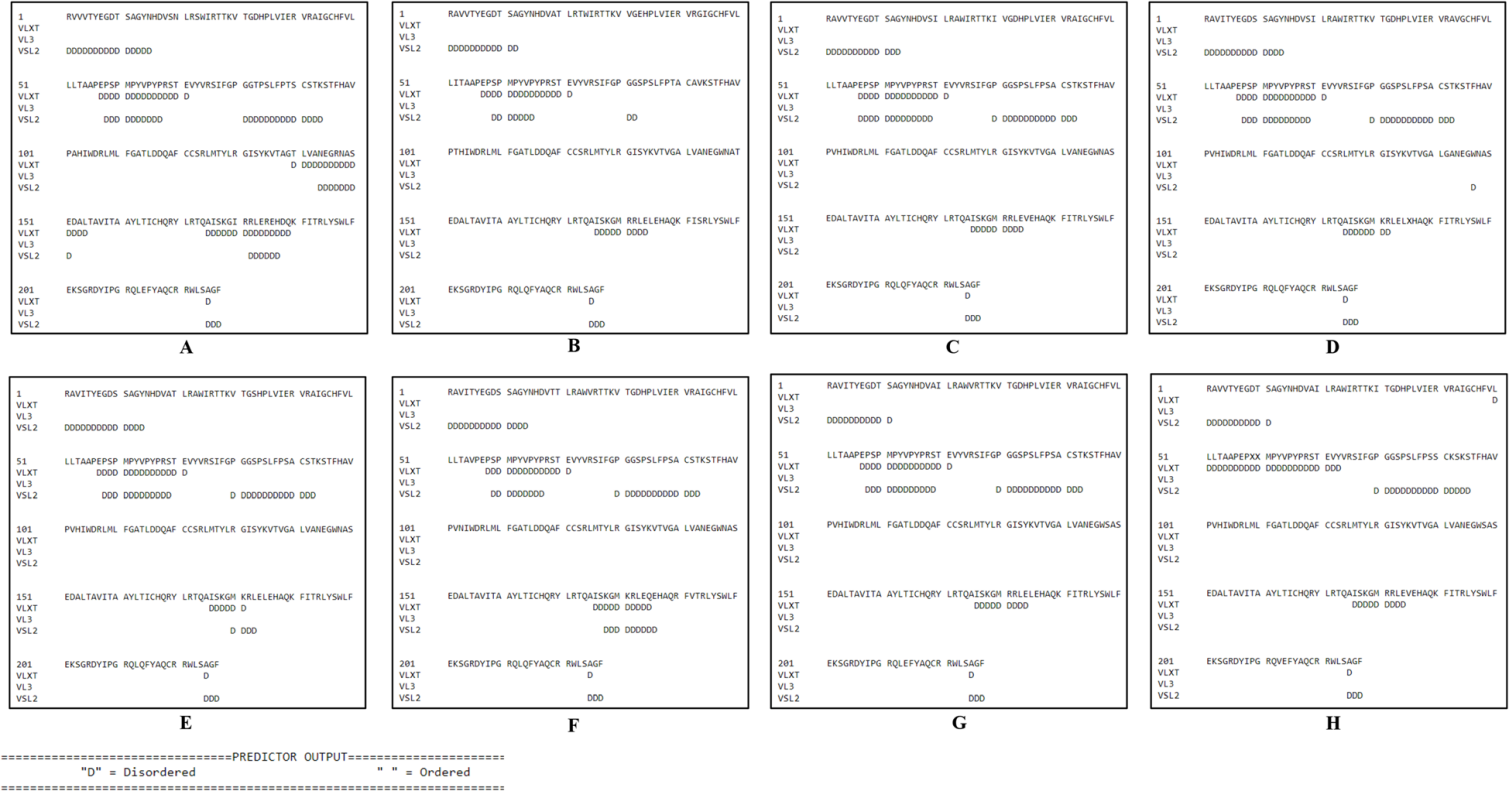
Prediction of disordered residues in HEV YDR. **A** JF443720 (GT 1); **B** M74506 (GT 2); **C** AB222182 (GT 3); **D** GU119961 (GT 4); **E** AB573435 (GT 5); **F** AB602441 (GT 6); KJ496143 (GT 7); and **H** KX387865 (GT 8). The prediction of disordered residues was carried out using three members of the family PONDR (Prediction of Natural Disordered Regions), i.e., VLXT, VL3, and VSL2. A threshold value of 0.5 was set to distinguish between ordered and disordered regions along the genome (dashed line). Regions above the threshold are predicted to be disordered. The predicted disordered residues are shown with the alphabet “D”

The webserver predicts the natural disordered regions upon single protein sequences. The resulting disorder profiles of YDR sequences with the predicted disorder scores clearly revealed them as moderately disordered proteins (Fig. 3A–H). They consisted of flexible N- and C-terminals with multiple flexible regions along the entire polypeptide chain length (Fig. 3A–H). The predicted intrinsic disordered residues obtained from three disorder predictors for YDR sequences are represented (Table 2) (Fig. 4A–H).

**Table 2:** The predicted percentage of intrinsic disorder scores of YDR in hepatitis E viruses

Predictor	Disordered regions	Overall percent disordered	Number of disordered regions	Longest disordered region	Average prediction score
**JF443720**					
**VLXT**	[57-71] EPSPMPYVPYPRSTE [140-154] TLVANEGRNASEDAL [175-189] AISKGIRRLEREHDQ [225-225] A	20.26	4	15	0.2573
**VSL2**	[1-15] RVVVTYEGDTSAGYN [58-67] PSPMPYVPYP [81-94] GGTPSLFPTSCSTK [144-151] NEGRNASE [182-187] RLEREH [225-227] AGF	100.00	6	15	0.3046
**M74506**					
**VLXT**	[57-71] EPSPMPYVPYPRSTE [176-184] ISKGMRRLE [225-225] A	11.01	3	15	0.1842
**VSL2**	[1-12] RAVVTYEGDTSA [59-65] SPMPYVP [81-82] GG [225-227] AGF	10.57	4	12	0.2586
**AB222182**					
**VLXT**	[57-71] EPSPMPYVPYPRSTE [176-184] ISKGMRRLE [225-225] A	11.01	3	15	0.1827
**VSL2**	[1-13] RAVVTYEGDTSAG [57-69] EPSPMPYVPYPRST [80-93] PGGSPSLFPSACST [225-227] AGF	18.94	4	14	0.2707
**GU119961**					
**VLXT**	[57-71] EPSPMPYVPYPRSTE [175-182] AISKGMR [225-225] A	10.57	3	15	0.1846
**VSL2**	[1-14] RAVVTYEGDTSAGY [58-69] PSPMPYVPYPRST [80-93] PGGSPSLFPSACST [147-147] W [225-227] AGF	19.38	5	14	0.3609
**AB573435**					
**VLXT**	[57-71] EPSPMPYVPYPRSTE [176-181] ISKGMK [225-225] A	9.69	3	15	0.1705
**VSL2**	[1-14] RAVVTYEGDTSAGY [58-69] PSPMPYVPYPRST [80-93] PGGSPSLFPSACST [180-183] MKRL [225-227] AGF	20.79	5	14	0.2823
**AB602441**					
**VLXT**	[58-71] PSPMPYVPYPRSTE [176-188] ISKGMKRLEQ [225-225] A	11.01	3	14	0.1905
**VSL2**	[1-14] RAVVTYEGDTSAGY [59-67] SPMPYVPYPR [80-93] PGGSPSLFPSACST [178-186] KGMKRLEQE [225-227] AGF	21.59	5	14	0.2859
**KJ496143**					
**VLXT**	[57-71] EPSPMPYVPYPRSTE [176-184] ISKGMKRRLE [225-225] A	11.01	3	15	0.1835
**VSL2**	[1-11] RAVVTYEGDTS [58-69] PSPMPYVPYPRST [80-93] PGGSPSLFPSACST [225-227] AGF	17.62	4	14	0.2746
**KX387865**					
**VLXT**	[50-73] LLLTAAPEPXXMPYVPYPRSTEVY [176-184] ISKGMKRRLE [225-225] A	14.98	3	24	0.2234
**VSL2**	[1-11] RAVVTYEGDTS [80-95] PGGSPSLFPSSCKSKS [225-227] AGF	13.22	3	16	0.2732

Note 1: The intrinsic disorder was not predicted in the YDR sequences using the VSL2

Note 2: JF443720 (GT 1); M74506 (GT 2); AB222182 (GT 3); GU119961 (GT 4); AB573435 (GT 5); AB602441 (GT 6); KJ496143 (GT 7); KX387865 (GT 8)

The individual YDR sequences were analyzed for the prediction of disordered regions. Based on the overall degree of intrinsic disorder, i.e., predicted fraction of disordered residues, the proteins are categorized into different intrinsic disorder variants: *structured proteins* (0–10%), *moderately disordered proteins* (10–30%), and *highly disordered proteins* (30–100%) [55, 56]. The percentage fraction of disordered residues was predicted in the range of 10–30%, by VLXT in combination with VSL2. The disorder profiles of the YDR sequences, obtained from disorder predictors (VLXT and VSL2), revealed them as moderately disordered proteins, as they consisted of 10–30% of the disordered residues in their polypeptide chain, with multiple flexible regions. It was observed that YDR sequences did not possess significant disorder as mostly it consisted of structured regions. Moreover, the absence of 30 or more consecutively long amino acid regions suggests a lack of long disordered regions in YDR sequences (Table 2). Figure 3 A–H represent the disorder profiles of YDR sequences obtained from three different predictors of the PONDR family. The graph profiles showed similarity in disorder in YDR sequences at both N- and C-terminals.

Thus, it was revealed that the presence of disordered residues in the conserved “LYSWLFE” counterpart in all the YDR sequences clearly indicated that this conserved motif was characterized by structural flexibility.

### Analysis of protein-binding propensity

**Fig. 5:**
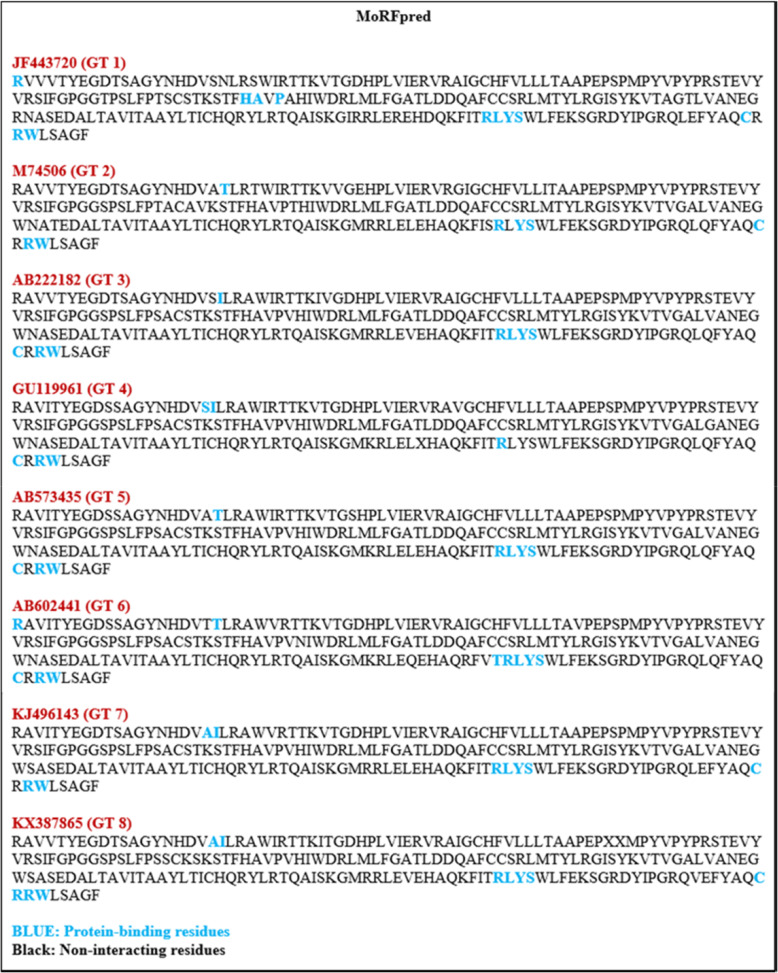
Analysis of protein-binding propensity of HEV YDR, i.e., JF443720 (GT 1), M74506 (GT 2), AB222182 (GT 3), GU119961 (GT 4), AB573435 (GT 5), AB602441 (GT 6), KJ496143 (GT 7), and KX387865 (GT 8). The resulting protein-binding profile was calculated using MoRFpred. YDR mainly contains MoRFs at C-terminals. The protein-binding residues are depicted in blue while the non-interacting residues are depicted in black

*MoRFpred*: The results of MoRFs (protein-binding regions) analysis are elaborated (Fig. 5), which clearly indicated that YDR had flexible C-terminals. These regions due to possession of MoRFs can be used for protein–protein interactions due to structural flexibility.

*DisoRDPbind*: DisoRDPbind did not predict the protein-binding residues within the YDR sequences.

Thus, the presence of MoRFs at N- and C-terminus in the YDR sequences indicated its involvement in interaction with the MTase and PCP domain for the ORF1 functionality respectively. Also, the MoRF presence in the conserved “LYSWLFE” counterpart in YDR sequences revealed its interactive role with the host cell receptor. Therefore, our protein-binding propensity analysis indicated the important role performed by YDR disorder in the functionality of these proteins.

### Analysis of nucleotide-binding propensity

A combination of different online predictors (DisoRDPbind, DRNApred, and PPRInt) was used to find out the situated protein residues that had propensity to bind to nucleotides (DNA and RNA).

#### Identification of RNA-binding regions

**Fig. 6:**
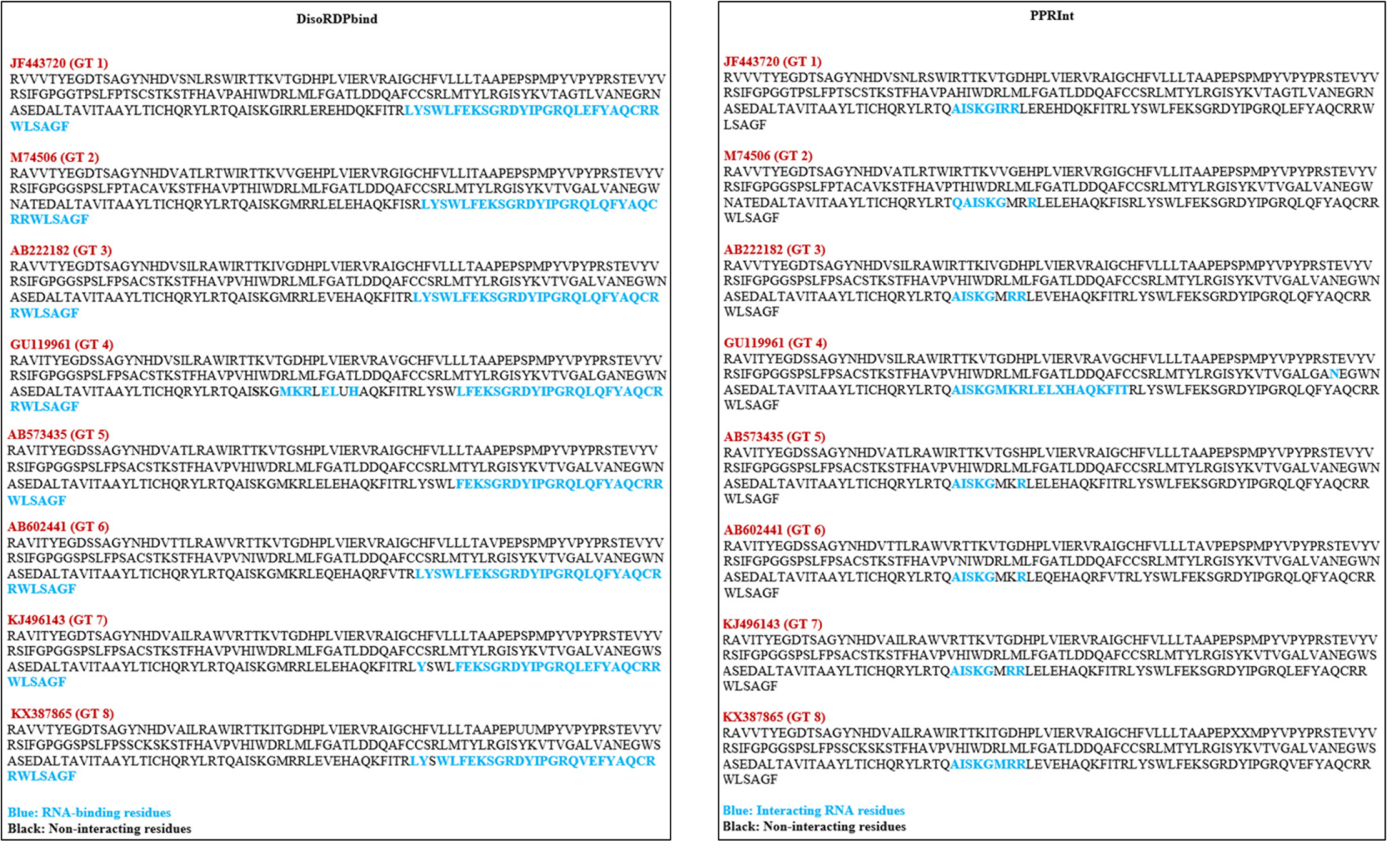
**A** Analysis of RNA-binding propensity of HEV YDR, i.e., JF443720 (GT 1), M74506 (GT 2), AB222182 (GT 3), GU119961 (GT 4), AB573435 (GT 5), AB602441 (GT 6), KJ496143 (GT 7), and KX387865 (GT 8). The resulting RNA-binding profile was calculated using webservers (A) DisoRDPbind and (B) PPRInt. The RNA-binding residues were situated at the C-terminus of the YDR. The identified RNA-binding residues are depicted in red while the non-interacting residues are depicted in black

*DisoRDPbind*: Several RNA-binding residues were identified at the C-terminus of the YDR sequences **(**Fig. 6A).

*DRNApred:* The RNA-binding residues were not predicted using the DRNApred server.

*PPRInt*: Numerous RNA-binding residues throughout the polypeptide chain of YDR sequences were identified (Fig. 6B).

Our RNA-binding propensity analysis revealed the presence of several RNA-binding residues in the YDR sequences. However, only the C-terminus residues in YDR showed RNA-binding affinity (as predicted by DisoRDPbind and PPRInt). Moreover, the residues were also identified within the highly conserved “LYSWLFE” segment (α-helix counterpart) of the YDR (predicted by DisoRDPbind and PPRInt).

#### Identification of DNA-binding regions

***DisoRDPbind***: The DNA-binding residues were found to be absent in the YDR sequences.

**Fig. 7:**
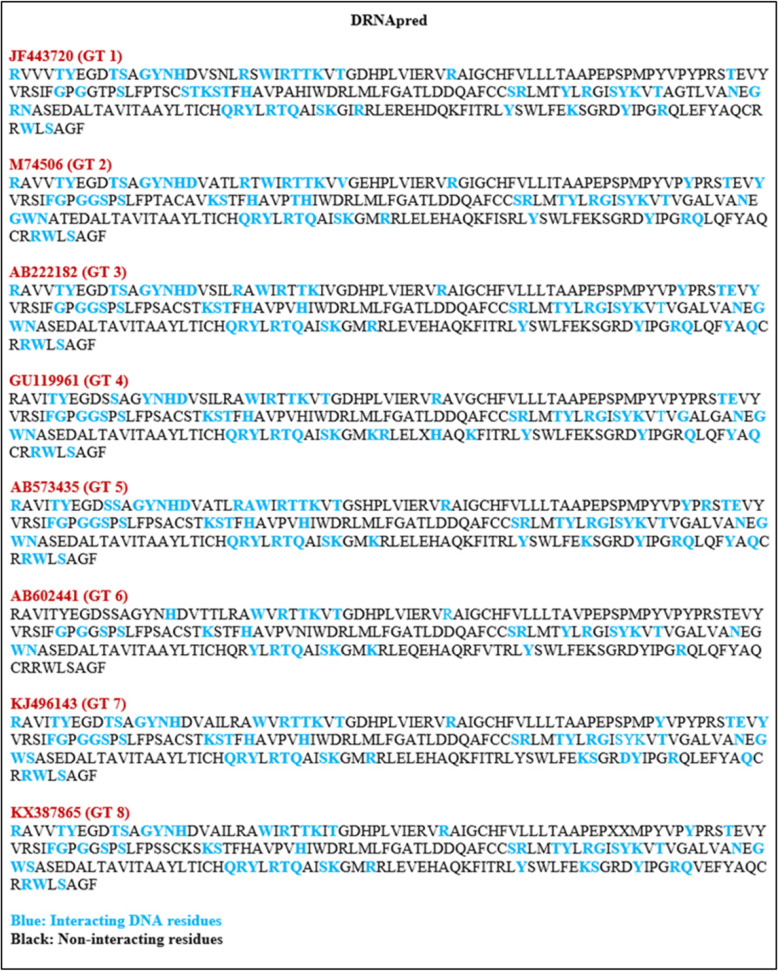
Analysis of DNA-binding propensity of HEV YDR, i.e., JF443720 (GT 1), M74506 (GT 2), AB222182 (GT 3), GU119961 (GT 4), AB573435 (GT 5), AB602441 (GT 6), KJ496143 (GT 7), and KX387865 (GT 8). The resulting DNA-binding profile was calculated using webservers DRNApred. The DNA-binding residues distributed throughout the polypeptide chains of the YDR sequences

***DRNApred***: The DNA-binding residues were observed at both the N- and C-terminals of the YDR sequences (Fig. 7).

Thus, our DNA-binding propensity analysis revealed the presence of several DNA-binding residues in the YDR sequences. Both the N- and C-terminals including the entire length of the polypeptide chain showed DNA-binding affinity towards YDR. Moreover, the residues were also identified within the highly conserved “LYSWLFE” segment (α-helix counterpart) of the YDR (as predicted by DRNApred).

Therefore, our nucleotide propensity analysis indicated the high propensities of these predicted residues towards RNA and DNA. Moreover, the residues predicted within the “LYSWLFE” segment indicated its involvement in the critical function of viral replication.

### Analysis of phosphorylation sites

**Fig. 8:**
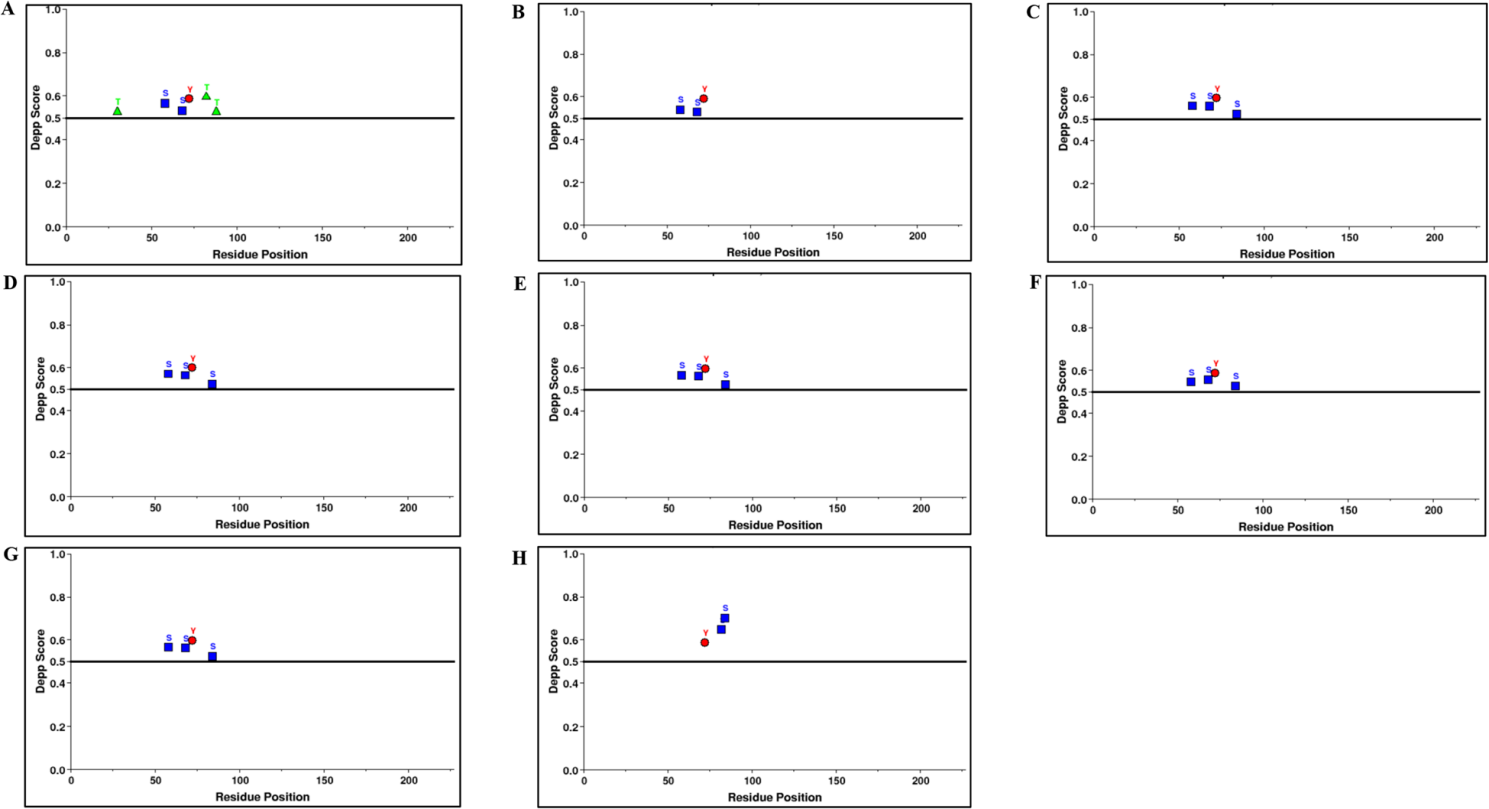
Prediction of phosphorylation sites showing the scores of phosphorylated residues (Ser, Thr, Tyr) along with the depicted scores within YDR. **A** JF443720 (GT 1); **B** M74506 (GT 2); **C** AB222182 (GT 3); **D** GU119961 (GT 4); **E** AB573435 (GT 5); **F** AB602441 (GT 6); KJ496143 (GT 7); and **H** KX387865 (GT 8). Graphs **A**–**H** represent the phosphorylation patterns of the YDR sequences of HEV. The score was computed using DEPP (Disorder Enhanced Phosphorylation Predictor). A threshold value of 0.5 was set to distinguish between ordered and disordered regions along the genome (line). The predicted phosphorylated residues above the threshold are represented as Ser (S), blue; Thr (T), green; and Tyr (Y), red

Our phosphorylation analysis showed the presence of phosphorylation sites (P-sites) in all the YDR sequences. The predicted phosphorylated residues, i.e., Ser, Thr, and Tyr, in HEV YDR sequences with the DEPP score are summarized (Table 3) (Fig. 8).

**Table 3:** Predicted number and percentage of phosphorylated residues in YDR of hepatitis E viruses

Sequences	Number of phosphorylated residues		
	Ser	Thr	Tyr
JF443720	2 out 17 (11.76%)	3 out of 20 (15.00%)	1 out of 12 (8.33%)
M74506	2 out 14 (14.28%)	0 out of 20 (0.00%)	1 out of 12 (8.33%)
AB222182	3 out of 17 (17.64%)	1 out of 12 (8.33%)	1 out of 12 (8.33%)
GU119961	3 out of 18 (16.66%)	0 out of 16 (0.00%)	1 out of 12 (8.33%)
AB573435	3 out of 18 (16.66%)	0 out of 17 (0.00%)	1 out of 12 (8.33%)
AB602441	3 out of 17 (17.64%)	0 out of 18 (0.00%)	1 out of 12 (8.33%)
KJ496143	3 out of 17 (17.64%)	0 out of 17 (0.00%)	1 out of 12 (8.33%)
KX387865	2 out 17 (11.76%)	0 out of 16 (0.00%)	1 out of 12 (8.33%)

**Fig. 9:**
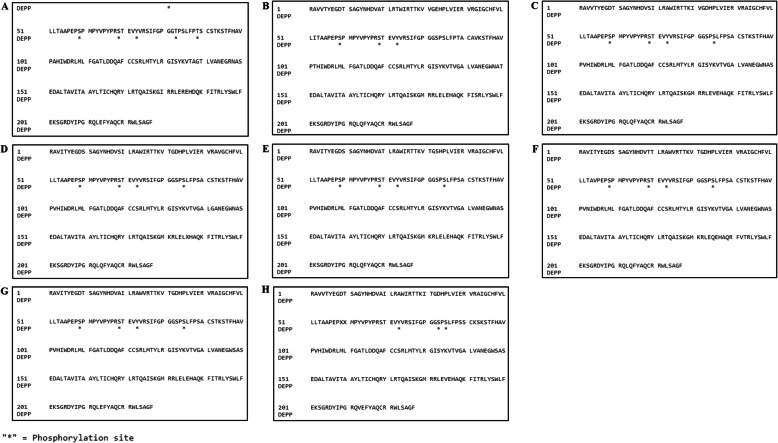
Depiction of phosphorylated residues within HEV YDR (**A**) JF443720 (GT 1); (**B**) M74506 (GT 2); (**C**) AB222182 (GT 3); (**D**) GU119961 (GT 4); (**E**) AB573435 (GT 5); (**F**) AB602441 (GT 6); KJ496143 (GT 7); and (**H**) KX387865 (GT 8). The was carried out using DEPP (Disorder Enhanced Phosphorylation Predictor). The predicted phosphorylated residues in the YDR proteins are marked with asterisk (*)

Our results revealed that Ser was found in higher fractions in comparison to the other phosphorylated residues, i.e., Thr and Tyr **(**Fig. 8A-H). It was revealed that most of the phosphorylation sites (P-sites) were found within intrinsically disordered regions of the YDR **(**S2A–H Figure). VLXT is considered the most accurate predictor due to the different attributes that make up this algorithm and good accuracy [57]. Thus, we used the disorder information (as predicted by VLXT) of YDR to correlate the presence of P-sites and non-phosphorylation sites. Figure 8A-H shows the phosphorylation pattern profiles of the YDR sequences with the predicted DEPP scores. Our results revealed that the phosphorylated residues (Ser, Thr, and Tyr) were present within the disordered fragments of YDR, which clearly indicated the correlation between disordered regions and phosphorylation sites (S2A–H Figure). The specific amino acid position of the predicted phosphorylated residues in YDR is shown (Fig. 9).

### Prediction of molecular functions

The putative molecular functions of the YDR based on the predicted 3D structures were identified using the COFACTOR algorithm. The consensus GO annotations associated to the models are summarized in Table 4.

**Table 4:** Predicted consensus GO terms for YDR models

Consensus GO terms	Description
**HEV**	
GO:0020037~heme binding	Interacting selectively and non-covalently with heme, any compound of iron complexed in a porphyrin (tetrapyrrole) ring
GO:0009055~electron transfer activity	Any molecular entity that serves as an electron acceptor and electron donor in an electron transport chain
GO:0004129~cytochrome-c oxidase activity	Catalysis of the reaction: 4 ferrocytochrome c + O2 + 4 H+ = 4 ferricytochrome c + 2 H2O
**Human**	
GO:0030598~rRNA N-glycosylase activity	Catalysis of the hydrolysis of the N-glycosylic bond at A-4324 in 28S rRNA from rat ribosomes or corresponding sites in 28S RNA from other species
**Swine**	
GO:0009486~cytochrome bo3 ubiquinol oxidase activity	Catalysis of the reaction: 2 ubiquinol + O2 + 4 H+ = 2 ubiquinone + 2 H2O + 4 H+ [periplasmic space]
GO:0020037~heme binding	Interacting selectively and non-covalently with heme, any compound of iron complexed in a porphyrin (tetrapyrrole) ring
GO:0005507~copper ion binding	Interacting selectively and non-covalently with copper (Cu) ions
GO:0048039~ubiquinone binding	Interacting selectively and non-covalently with ubiquinone, a quinone derivative with a tail of isoprene units
GO:0000166~nucleotide binding	Interacting selectively and non-covalently with a nucleotide, any compound consisting of a nucleoside that is esterified with (ortho)phosphate or an oligophosphate at any hydroxyl group on the ribose or deoxyribose
GO:0016682~oxidoreductase activity	Catalysis of an oxidation-reduction (redox) reaction in which a diphenol, or related compound, acts as a hydrogen or electron donor and reduces oxygen
GO:0015453~-driven active transmembrane transporter activity	Primary active transport of a solute across a membrane, driven by the exothermic flow of electrons from a reduced substrate to an oxidized substrate
GO:0009055~electron transfer activity	Any molecular entity that serves as an electron acceptor and electron donor in an electron transport chain
GO:0015078~proton transmembrane transporter activity	Enables the transfer of a proton from one side of a membrane to the other
GO:0018662~phenol 2-monooxygenase activity	Catalysis of the reaction: phenol + NADPH + H+ + O2 = catechol + NADP+ + H2O
**Wild boar**	
GO:0004096~catalase activity	Catalysis of the reaction: 2 hydrogen peroxide = O2 + 2 H2O.
GO:0020037~heme binding	Interacting selectively and non-covalently with heme, any compound of iron complexed in a porphyrin (tetrapyrrole) ring
GO:0016874~ligase activity	Catalysis of the joining of two molecules, or two groups within a single molecule, using the energy from the hydrolysis of ATP, a similar triphosphate, or a pH gradient
GO:0050242~pyruvate, phosphate dikinase activity	Catalysis of the reaction: ATP + phosphate + pyruvate = AMP + diphosphate + 2 H(+) + phosphoenolpyruvate
GO:0005524~ATP binding	Interacting selectively and non-covalently with ATP, adenosine 5′-triphosphate, a universally important coenzyme and enzyme regulator
GO:0016301~kinase activity	Catalysis of the transfer of a phosphate group, usually from ATP, to a substrate molecule
GO:0004108~citrate (Si)-synthase activity	Catalysis of the reaction: acetyl-CoA + H2O + oxaloacetate = citrate + CoA, where the acetyl group is added to the si-face of oxaloacetate; acetyl-CoA thus provides the two carbon atoms of the pro-S carboxymethyl group
**Camel**	
GO:0043167~ion binding	Interacting selectively and non-covalently with ions, charged atoms, or groups of atoms

The molecular functions included heme binding, copper ion binding, ubiquinone binding, nucleotide binding, ATP binding, ion binding, electron transfer activity, cytochrome-c oxidase activity, N-glycosylase activity, ligase activity, kinase activity, and citrate-synthase activity. Thus, binding interactions and catalytic activities were the major functional roles that were attributed to YDR in respective hosts. The binding interactions, such as heme binding (GO:0020037), ion binding (GO:0043167), and nucleotide binding (GO:0000166), revealed the propensity of YDR to bind to a variety of molecules (protein, nucleotide, ion), similar to our earlier results. Furthermore, the predicted different catalytic activities, such as electron transfer activity (GO:0009055) and cytochrome c oxidase activity (GO:0004129), revealed the significant mitochondrial functional roles associated with YDR in respective host organisms (Table 4).

## Discussion

The functional implication of YDR in HEV adaptation remains to be explored. To complete the life cycle, viruses require various interactions with the components of the host cells, beginning from the virus’s attachment, its entry, commandeering the host machinery, synthesis of the viral components, and particle assembly to the last phase, i.e., exiting as new infectious particles from the host cell [58]. All these stages rely heavily on the intrinsic disorder prevalent in viral proteins [58]. Thus, intrinsic disorder is linked with the pathogenesis and infection of the virions. Therefore, the presented study reports the analysis on the unstructured regions of YDR to shed novel light on its functionality in HEV regulation. Moreover, other parameters in proteins such as structural annotation, function, and protein–protein interactions also influence the process of adaptation [59]. Thus, we employed different bioinformatics predictors based on a set of algorithms to analyze the effect of these factors on YDR in order to delineate its role in viral adaptation.

The diversifications in structure and amino acid composition play a vital role in the evolutionary adaptation. Our initial structural investigation on the YDR model revealed the presence of all three secondary structural components, i.e., alpha-helix (α), beta-strand (β), and coils. All the YDR sequences consisted of higher percentage of α-helices as compared to β-strands with the predominance of coils which is in agreement with the recent study [60]. Then, we next examined the amino acid composition in different YDR sequences to reveal the residue percentages. The disordered regions are rooted in the idiosyncrasies of their amino acid composition, which are deficient in order-promoting residues (Trp, Cys, Tyr, Ile, Phe, Val, Asn, and Leu) and abundant in disorder-promoting residues (Arg, Pro, Gln, Gly, Glu, Ser, Ala, and Lys) [48–54]. Thus, sequence-based analyses of YDR uncovered both ordered (Val, Leu, Phe, Tyr, and Ile) and disordered (Arg, Ala, Ser, Pro, Gly) promoting residues, categorizing it as DP fragments, i.e., consisting of both structured (ordered) and unstructured (disordered) regions [47]. These DP fragments exhibit peculiar characteristics between order and disorder which distinguish them from both regularly folded proteins and intrinsically disordered proteins/ protein fragments. Additionally, Dunker and colleagues demonstrated the dominance of six signature amino acids (Thr, Arg, Gly, Asn, Pro, and Asp) in DP fragments which determine their distinguishing conformational physiognomies. Thus, predominance of hydrophobic amino acid residues such as Thr, Arg, Gly, and Pro further substantiates our present findings that YDR possesses the characteristics of “Dual Personality” (DP) fragments [47].

In line with this, our intrinsic disorder propensity analysis also revealed YDR to be moderately disordered proteins. Based on the overall degree of intrinsic disorder, i.e., predicted fraction of disordered residues, the different intrinsic disorder variants are categorized into structured proteins (0–10%), moderately disordered proteins (10–30%), and highly disordered proteins (30–100%) [55, 56]. The YDR sequences considered in the study consisted of 10–30% of the disordered residues and thus were categorized into moderately disordered proteins, i.e., protein hybrids consisting of both structured regions as well as unstructured (disordered) regions. Thus, it is noteworthy to mention that YDR possessed both ordered and disordered domains [47]. Additionally, evidence has suggested order/disorder transitions in some DP fragments (upon signals), which can contribute to protein activity through regulation [47]. Our intrinsic disorder propensity analysis suggested the presence of some disordered regions in YDR sequences (found to be ordered in other databases), which suggests their order to disorder tendency upon binding. This clearly reveals the peculiar characteristic of dual-personality fragments which straddles between the ordered and disordered protein phases [61, 62]. Additionally, highly flexible and disordered segments in DP on binding with substrate or by protein phosphorylation become ordered fragments, suggesting order/disorder transition in DP fragments [47]. This substantiates our findings which revealed the role of YDR in regulation through order/disorder tendency.

Furthermore, it has been well documented that disordered protein segments possess enormous flexibility [34]. These intrinsically disordered segments in proteins perform a variety of important cellular functions by binding through specific interactions with RNA, DNA, and protein ligands [35, 36]. There are many computational methods through which intrinsically disordered proteins (IDPs) or intrinsically disordered protein regions (IDPRs) can be predicted within protein sequences; however, only few of them can predict the given protein’s functions through its protein-binding propensity. MoRFpred is a computational sequence-based prediction tool used to characterize short disorder-to-order transition binding regions in the target protein upon identification. It is based on a novel design and identifies all types of MoRFs (α, β, coil, and complex) with accuracy [41]. DisoRDPbind webserver predicts the disordered RNA-, DNA-, and protein-binding residues located within the disordered segments of target proteins. We used DisoRDPbind as it is user-friendly and provides accurate predictions, as well as it provides insights into the multiple functions carried out by the disordered protein regions [42]. Moreover, protein–RNA and protein–DNA interactions also play diverse and essential cellular functional roles [35, 36]. Most of the sequence-based bioinformatic predictor tools are relatively slow and could not accurately predict the RNA- and DNA-binding residues and sometimes result in cross-predictions of RNA-binding residues with DNA-binding residues and vice versa. Therefore, we used DRNApred, a relatively fast sequence-based method, that accurately predicts and differentiates RNA- and DNA-binding residues [43]. Therefore, we used a combination of different predictors (MoRFpred, DisoRDPbind, and DRNApred) to identify the disorder-based functions of YDR by carrying out its sequence-based binding tendency.

MoRFs specifically focus on interactions between proteins and are considered as a specific subset of DP fragments [47]. MoRFs are short-disordered segments in IDPs/IDPRs that are prone to interactions with their binding partners upon transition from a disorder-to-order state [44]. The presence of MoRFs at the C-terminals of YDR suggests its engagement with the ORF1 PCP domain. Also, MoRF at N-terminus in two YDR sequences (JF443720 and AB602441), suggests that YDR is engaged in with the MTase domain. The sequence alignment of the HEV and the closely related viruses (EEV, SFV, and SINV) showed universally conserved residues (Lys, Ser, and Trp) in the amphipathic α-helical segment (LYSWLFE), which has been implicated in intracellular membrane binding. Similarly, the YDR of nonstructural ORF1 polyprotein consists of a membrane-binding motif having structural/functional significance in the replication and infection of HEV [30]. The multiple sequence alignment of the HEV strains showed the presence of a highly conserved α-helix segment (LYSWLFE) within the YDR of ORF1. This highly conserved α-helical motif in YDR of HEV plays an indispensable role in membrane-binding interaction. Moreover, Trp, a hydrophobic residue, within this conserved segment has been demonstrated to play a crucial role in PPIs through protein folding. Thus, the presence of disordered residues in the conserved “LYSWLFE” counterpart clearly suggests that this conserved motif is essential for the interaction of YDR with their binding partners due to the possession of structural flexibility. Additionally, the presence of MoRFs in this conserved region n YDR further signifies that these conserved residues might assist in guiding the specific function of membrane binding. Therefore, it is interesting to mention that our MoRFs prediction in this signature α-helical counterpart provides compelling evidence of YDR involvement in membrane binding through PPI. Furthermore, we also predicted the interactions between protein and DNA- and RNA-binding residues to provide deep knowledge into the functional role of YDR. Our nucleotide-binding analysis revealed that YDR showed high propensity towards RNA- and DNA-binding residues. Identification of nucleotide-binding residues at C-terminals, which also included some residues within the LYSWLFE segment in the YDR (as predicted by DisoRDPbind), revealed flexible (disorder-based) RNA-binding regions, thus elucidating the critical residue role in viral replication of HEV as suggested earlier [30]. Moreover, the presence of both RNA- and DNA-binding residues within the conserved “LYSWLFE” segment revealed that these residues may play an important role at the transcriptional or translational level which is in accordance with the previous report [30]. Thus, the presence of molecular recognition (protein, RNA, and DNA-binding) in the LYSWLFE conserved counterpart (C-terminus) suggests YDR functional/structural essentiality in HEV replication and intracellular membrane binding which is consistent with the previous report [30]. Though these findings enhance our knowledge on this precisely understood Y-domain, however, further information is still required to delineate its function and its conserved residues criticality in the viral replication.

Fluctuation in the conformation of the intrinsically disordered regions in proteins transiently reveals dynamic interaction motifs, which lead to post-translational modifications (PTMs), resulting in their interaction with several target protein molecules that have an effect on cell cycle control [63, 64]. PTM is an essential requirement of a protein to carry out the regulation of various functions. Phosphorylation of viral proteins for many acute RNA viruses including Alphaviruses [65–68] and Flaviviruses [69–73] has been demonstrated to be critical for protein functionality. Protein phosphorylation is also essential for many intracellular pathogens to establish a productive infection cycle [74, 75]. Also, phosphorylation is required for protein folding, signal transduction, intracellular localization PPIs, transcription regulation, cell cycle progression, survival, and apoptosis [76]. Thus, the phosphorylation patterns of YDR were analyzed to study its related functions using an online algorithm DEPP. It was revealed that all the YDR sequences consisted of P-sites. Our observations revealed that P-sites were predicted within the disordered regions of the YDR’s polypeptide chains, suggesting tight interconnection between protein phosphorylation and disordered YDR regions. These findings are in accordance with the existing literature which suggested that the overall phosphorylated residues show an inclination towards disordered regions rather than the ordered protein segments [77, 78]. Indeed, computational analysis through various prediction tools has shown that disordered protein segments are enriched in phosphorylation sites (P-sites) [77, 78]. This underlines the significance of disordered regions as display sites for PTMs, probably due to the conformational flexibility provided to the display sites by the disordered region over ordered region in proteins [79, 80]. Furthermore, DP fragments have been closely linked to post-translational modifications, as post-translationally modified sites are located at/close to DP segments [47], further signifying that YDR has the characteristics of a DP molecule [47]. Moreover, the hydroxyl group present in the disordered protein segments of serine has been suggested as a target for phosphorylation by protein kinases [81]. Thus, the higher number of the predicted phosphorylated serine residues in the YDR sequences reveals the flexibility and interacting ability, characterizing its important role in protein regulation via various biological processes [47].

Furthermore, the predicted YDR 3D models were used to predict the molecular functions using GO annotations [35, 36]. The molecular functional roles revealed numerous potential sites. The predicted sites were shown to have interacted with several ligands including modified sites that bind to enzymes in conjunction with sites binding to nucleotides, proteins, and metal ions. Thus, our results suggest the involvement of YDR in binding to a wide range of substrates. These types of interactions have been reported to contribute to the regulation of various processes in cells such as cellular signal transduction, phosphorylation, transcription, and translation [34]. Moreover, the multiple catalytic functions associated with YDR in different hosts clearly indicate the YDR multifunctionality associated with it. Electron transfer activity and cytochrome c oxidase activity were among major catalytic functions, thus revealing YDR involvement in HEV regulation as mitochondria not only serve as signaling hubs for immune responses but also lead to facilitation of downstream signaling resulting in IFN synthesis [82, 83]. Mitochondrian remains in constant communication with the cytosol for the initiation of biological events. Additionally, mitochondrial functions are also strategically altered by viruses which affect the energy production, metabolism, and immune signaling [84]. Moreover, it has been suggested that complex III of the mitochondrial electron transport chain performs diverse biological functions [85, 86]. Recently, a study has suggested the important role of complex III in HEV infection [87]. It has been demonstrated that inhibition of complex III inhibits the replication of HEV, i.e., complex III is required for the sustenance of HEV infection [87]. These findings provide further evidence that YDR participates in regulatory functions of HEV.

Moreover, several disordered regions in nonstructural proteins have been demonstrated to play specific regulatory functions in viruses [88]. For instance, replication of hepatitis C virus (HCV) depends on the nonstructural NS5A protein which forms a multi-protein complex [89] by interacting with numerous viral and host proteins [90, 91] via its disordered domain [92]. The disordered region in the P (Polymerase) protein has been reported in Paramyxoviridae [93–95] and Rhabdoviridae [96, 97] at its N-terminus (PNT). It has been demonstrated that PNT domain interacts with N (nucleocapsid) and cellular protein of MeV (Measles virus) and N and L proteins of SeV (Sendai virus). The disordered N in the MeV has been shown to interact with several viral and host cellular components [98]. The disordered components along with the structural components were also observed in proteins like nucleoprotein and phosphoprotein of Nipah and Hendra viruses [99]. These protein–protein interactions result in the occurrence of several significant biological functions. Moreover, the polyproline region (PPR) of nonstructural ORF1 has been associated with the regulation of HEV in addition to its role in replication, due to its characteristic intrinsic disorder property [100]. Thus, it is noteworthy to mention that the intrinsic disordered regions in YDR could perform crucial regulatory functions by interacting with the other viral and host components.

To sum up our observations, it can be hypothesized that YDR has regulatory functions in addition to its role in the replication of HEV that is essential for viral adaptation. The inclusive information provided in this prospective study thus strongly proposes the role of YDR in HEV adaptation.

## Conclusions

The current study provides novel data on the role of YDR in HEV adaptation. The amino acid distribution revealed the signature residues prevalent in DP fragments. The presence of both ordered and disordered amino acid residues revealed YDR as protein hybrids. The occurrence of the unstructured region in YDR sequences suggested their disorder and flexibility. We also established that all the YDR sequences consisted of MoRFs, thus revealing its disorder-based propensity towards protein-binding partners. Furthermore, identification of several RNA- and DNA-binding sites in the YDR sequences suggested its critical role in the interaction with the hosts and further viral infection. The presence of various phosphorylation sites in YDR further signified it as an important constituent of mechanisms involving cellular and signaling pathways. Additionally, the presence of P-sites within the disordered segments of YDR further substantiated our findings, as PTM sites are located at/close to DP fragments. Furthermore, structure-based analysis of YDR models revealed several potential sites which further signifies their role in vital processes like cellular signaling transduction, phosphorylation, transcription, and translation by interacting with several ligand molecules, which suggested its noteworthy multiple functions associated with it. The involvement of YDR in mitochondrial functions further revealed its association with regulatory functions. Due to the DP flexibility to associate with different physiological partners, our analysis is envisaged to assist in producing important knowledge in the interaction of YDR with other HEV proteins. Furthermore, delineations of these interactions could possibly contribute to future research in revealing the molecular biology of HEV.

## Supplementary Information

**Additional file 1 : S1 Figure (A).** Generated 3D models of the HEV YDR. (A) JF443720 (GT 1); (B) M74506 (GT 2); (C) AB222182 (GT 3); (D) GU119961 (GT 4); (E) AB573435 (GT 5); (F) AB602441 (GT 6); KJ496143 (GT 7); and (H) KX387865 (GT 8). The prediction was carried out using Phyre2.

**Additional file 2 : S2 Figure.** Correlation between disordered and phosphorylated residues within HEV YDR (A) JF443720 (GT 1); (B) M74506 (GT 2); (C) AB222182 (GT 3); (D) GU119961 (GT 4); (E) AB573435 (GT 5); (F) AB602441 (GT 6); KJ496143 (GT 7); and (H) KX387865 (GT 8). The prediction of disordered residues was carried out using three members of the family PONDR (Prediction of Natural Disordered Regions), i.e., VLXT, VL3 and VSL2. The specific amino acid position of the prediction phosphorylated residue was carried out using DEPP (Disorder Enhanced Phosphorylation Predictor). The predicted disordered residues are shown with alphabet ‘D’ while the predicted phosphorylated residues in the YDR proteins are marked with asterisk (*). This suggests that the phosphorylated residues are present within the disordered regions of YDR.

**Additional file 3 : S1 Table.** List of HEV YDR sequences analyzed in the present study

## Data Availability

Not applicable

## Abbreviations

HEV: Hepatitis E virus
YDR: Y-domain region
GT: Genotype
O: Ordered
D: Disordered
DP: Dual personality
MoRFs: Molecular recognition features
PONDR: Predictor of natural disordered regions
PPRInt: Prediction of protein RNA-interaction
