# Supplementary figures and images for: Role of “dual-personality” fragments in HEV adaptation—analysis of Y-domain region
Source: J Genet Eng Biotechnol. 2021 Oct 12;19:154. doi: 10.1186/s43141-021-00238-8 (PMC8511232; doi:10.1186/s43141-021-00238-8)

## Slide 1
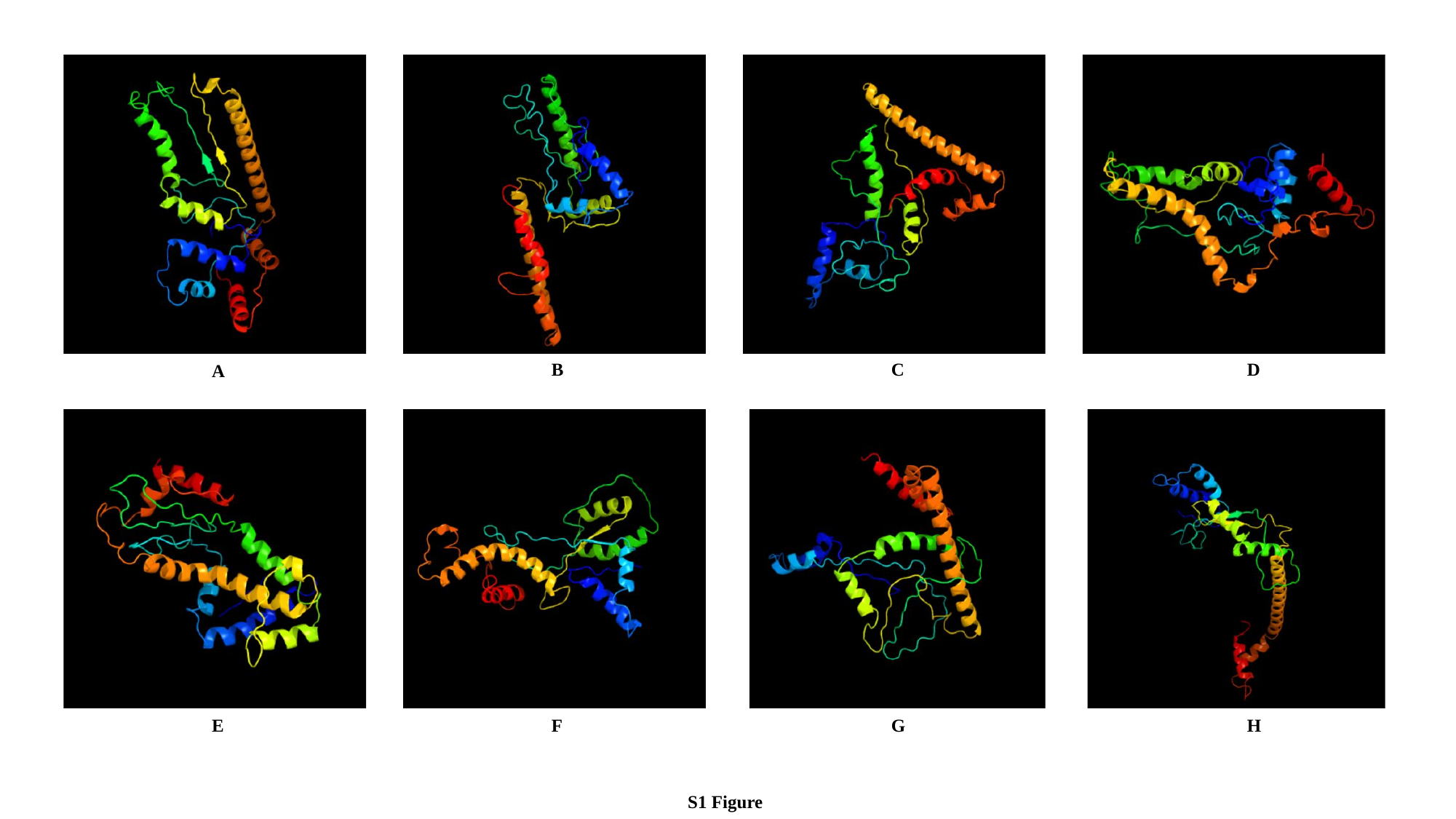

D
B
C
A
H
G
F
E
S1 Figure

Supplement: Supplementary file 1 — Additional file 1 : S1 Figure (A). Generated 3D models of the HEV YDR. (A) JF443720 (GT 1); (B) M74506 (GT 2); (C) AB222182 (GT 3); (D) GU119961 (GT 4); (E) AB573435 (GT 5); (F) AB602441 (GT 6); KJ496143 (GT 7); and (H) KX387865 (GT 8). The prediction was carried out using Phyre2. [file 43141_2021_238_MOESM1_ESM.pptx]

## Slide 1
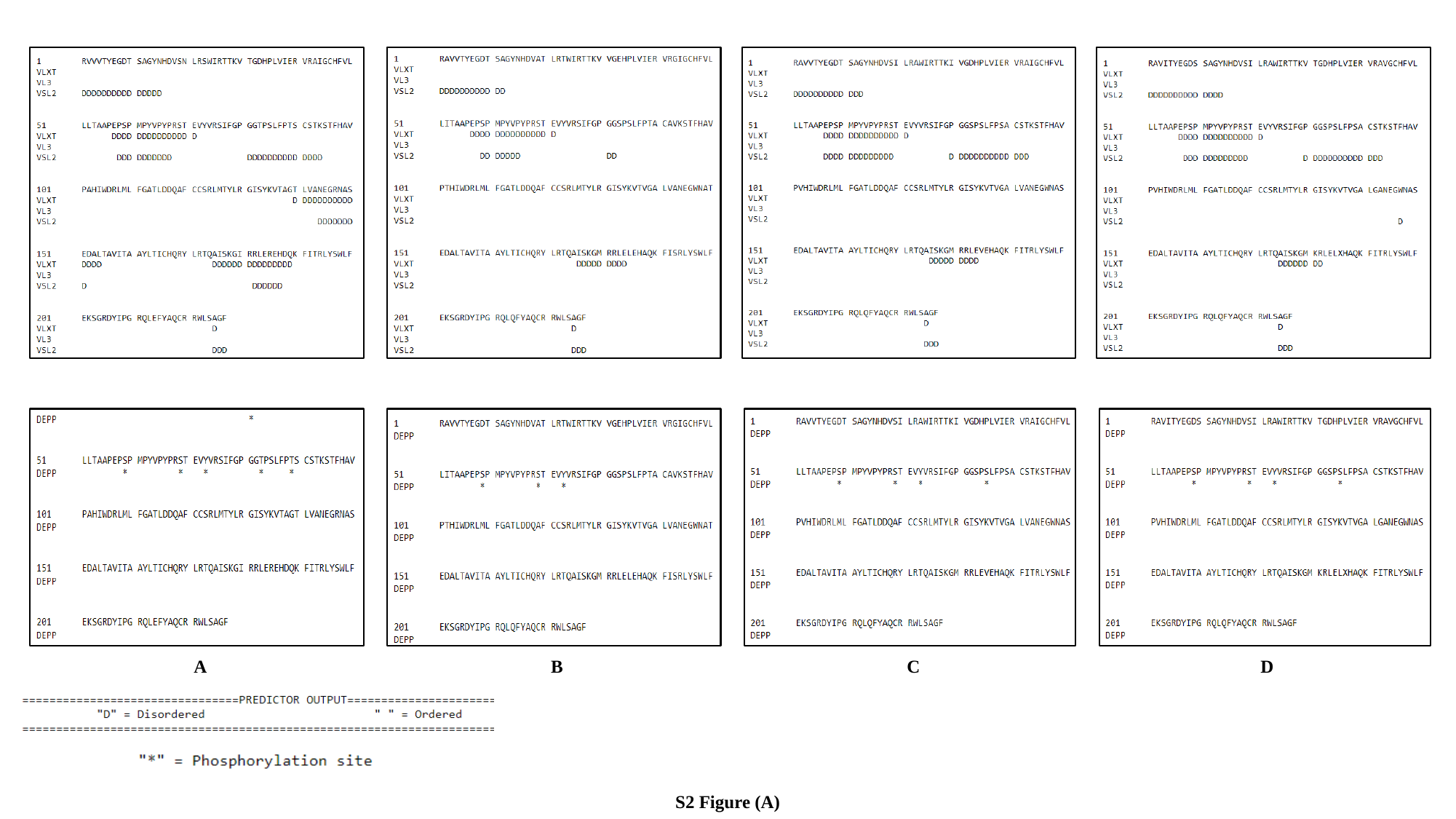

B
C
D
A
S2 Figure (A)

Supplement: Supplementary file 2 — Additional file 2 : S2 Figure. Correlation between disordered and phosphorylated residues within HEV YDR (A) JF443720 (GT 1); (B) M74506 (GT 2); (C) AB222182 (GT 3); (D) GU119961 (GT 4); (E) AB573435 (GT 5); (F) AB602441 (GT 6); KJ496143 (GT 7); and (H) KX387865 (GT 8). The prediction of disordered residues was carried out using three members of the family PONDR (Prediction of Natural Disordered Regions), i.e., VLXT, VL3 and VSL2. The specific amino acid position of the prediction phosphorylated residue was carried out using DEPP (Disorder Enhanced Phosphorylation Predictor). The predicted disordered residues are shown with alphabet ‘D’ while the predicted phosphorylated residues in the YDR proteins are marked with asterisk (*). This suggests that the phosphorylated residues are present within the disordered regions of YDR. [file 43141_2021_238_MOESM2_ESM.zip › Figure S2AR1.pptx]

## Slide 1
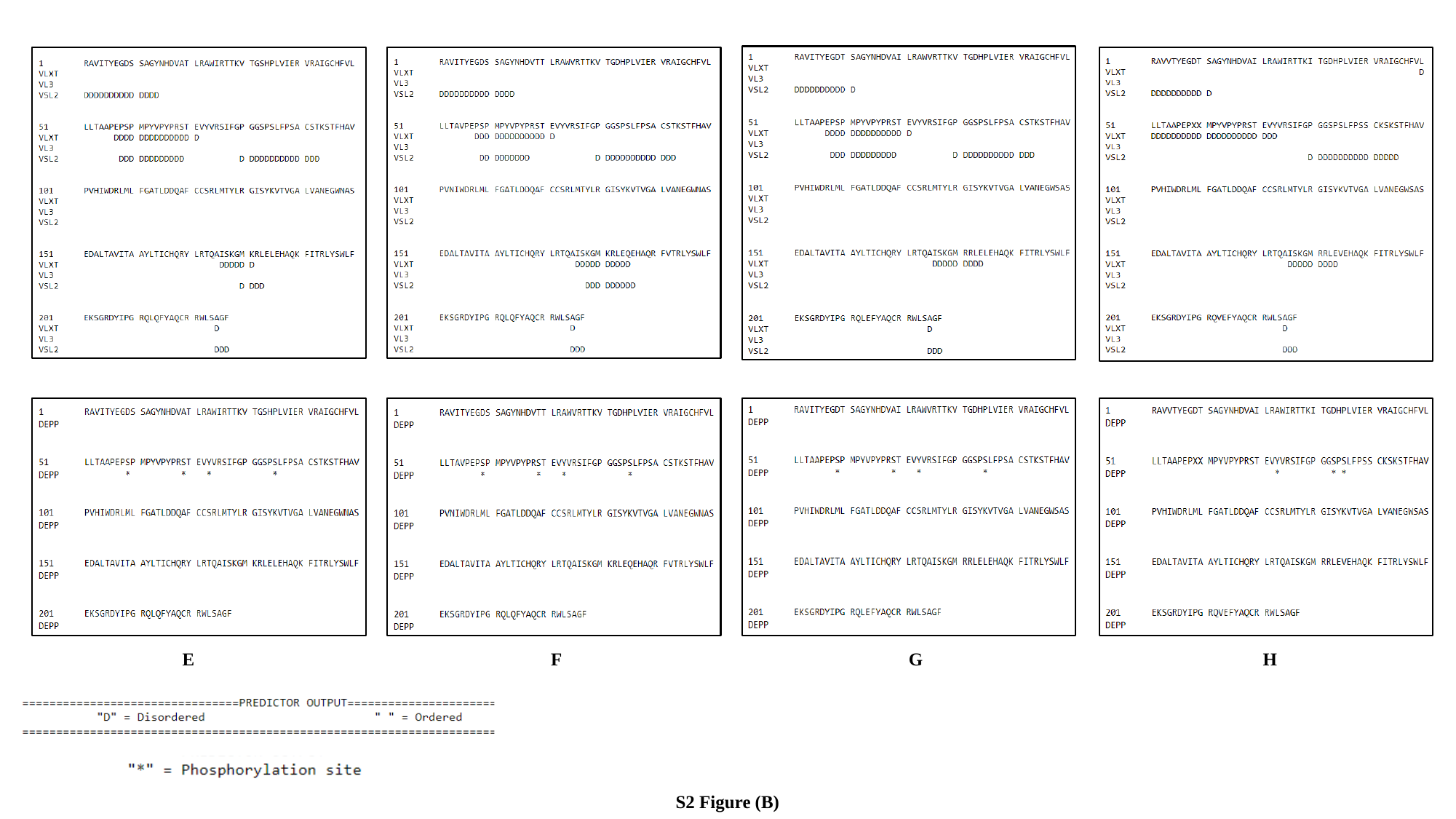

H
F
G
E
S2 Figure (B)

Supplement: Supplementary file 2 — Additional file 2 : S2 Figure. Correlation between disordered and phosphorylated residues within HEV YDR (A) JF443720 (GT 1); (B) M74506 (GT 2); (C) AB222182 (GT 3); (D) GU119961 (GT 4); (E) AB573435 (GT 5); (F) AB602441 (GT 6); KJ496143 (GT 7); and (H) KX387865 (GT 8). The prediction of disordered residues was carried out using three members of the family PONDR (Prediction of Natural Disordered Regions), i.e., VLXT, VL3 and VSL2. The specific amino acid position of the prediction phosphorylated residue was carried out using DEPP (Disorder Enhanced Phosphorylation Predictor). The predicted disordered residues are shown with alphabet ‘D’ while the predicted phosphorylated residues in the YDR proteins are marked with asterisk (*). This suggests that the phosphorylated residues are present within the disordered regions of YDR. [file 43141_2021_238_MOESM2_ESM.zip › Figure S2BR1.pptx]
