# Supplementary material for: Role of “dual-personality” fragments in HEV adaptation—analysis of Y-domain region
Source: J Genet Eng Biotechnol. 2021 Oct 12;19:154. doi: 10.1186/s43141-021-00238-8 (PMC8511232; doi:10.1186/s43141-021-00238-8)
Supplement: Supplementary file 3 — Additional file 3 : S1 Table. List of HEV YDR sequences analyzed in the present study [file 43141_2021_238_MOESM3_ESM.docx]

**S1 Table** List of HEV YDR sequences analyzed in the present study

| **No** | **Sequence Accession ID** | **Genotype (G)** | **Host** |
| --- | --- | --- | --- |
| 1 | AF444002 | **-** | **-** |
| 2 | JF443720 | I | Homo sapiens |
| 3 | M74506 | II | - |
| 4 | AB222182 | III | Wild boar |
| 5 | GU119961 | IV | Swine |
| 6 | AB573435 | V | *Sus scrofa leucomystax* |
| 7 | AB602441 | VI | *Sus scrofa leucomystax* |
